# Virological failure rates and HIV-1 drug resistance patterns in patients on first-line antiretroviral treatment in semirural and rural Gabon

**DOI:** 10.7448/IAS.15.2.17985

**Published:** 2012-11-28

**Authors:** Florian Liégeois, Caroline Vella, Sabrina Eymard-Duvernay, Jeanne Sica, Laurent Makosso, Augustin Mouinga-Ondémé, Arnaud Delis Mongo, Vanina Boué, Christelle Butel, Martine Peeters, Jean-Paul Gonzalez, Eric Delaporte, François Rouet

**Affiliations:** 1 Laboratoire de Rétrovirologie, CIRMF, BP769, Franceville, Gabon; 2 UMI 233 «Trans VIH MI» (Transitions épidémiologiques, recherches translationnelles appliquées au VIH et aux Maladies Infectieuses), Institut de Recherche pour le Développement (IRD), UCAD, Université de Montpellier 1, Montpellier, France;; 3 Centre de Traitement Ambulatoire (CTA), Franceville, Gabon; 4 Centre de Traitement Ambulatoire (CTA), Koulamoutou, Gabon

**Keywords:** HIV, Africa, antiretroviral therapy, viral load, resistance

## Abstract

**Introduction:**

As antiretroviral treatment (ART) continues to expand in resource-limited countries, the emergence of HIV drug resistance mutations (DRMs) is challenging in these settings. In Gabon (central Africa), no study has yet reported the virological effectiveness of initial ART given through routine HIV care.

**Methods:**

Following the World Health Organization (WHO) recommendations, a cross-sectional study with a one-time HIV-1 RNA viral load (VL) measurement was conducted in Gabon to assess virological failure (VF) defined by a VL result ≥1000 copies/ml and DRMs among adult patients living with non-B HIV-1 strains and receiving first-line non-nucleoside reverse transcriptase inhibitor (NNRTI)-based antiretroviral therapy for at least 12 months. Risk factors associated with VF and DRMs were assessed.

**Results:**

Between March 2010 and March 2011, a total of 375 patients were consecutively enrolled from two decentralized (one semirural and one rural) HIV care centres. Median time on ART was 33.6 months (range, 12–107). Overall, the rate of VF was 41.3% (36.4–46.4). Among viremic patients, 56.7% (80/141) had at least one DRM and 37.6% had dual-class resistance to nucleoside reverse transcriptase inhibitors (NRTIs) and NNRTIs. The most frequent DRMs were K103N/S (46.1%) and M184V/I (37.6%). Thymidine analogue mutations were found in 10.6%. Independent risk factors associated with VF were being followed up at the semirural centre (*P*=0.033), having experienced unstructured treatment interruptions (*P*=0.0044), and having low CD4^+^ counts at enrolment (*P*<0.0001). A longer time on ART (*P*=0.0008) and being followed up at the rural centre (*P*=0.021) were risk factors for DRMs.

**Conclusions:**

This is the first study conducted in Gabon providing VF rates and DRM patterns in adult patients receiving first-line ART. In sub-Saharan Africa, where NNRTI-based regimens are recommended as the standard for first-line ART, strengthening virological monitoring together with preventing unplanned treatment interruptions are a global public health priority.

## Introduction

Over the past decade, the roll-out of antiretroviral therapy (ART) has dramatically increased in low- and middle-income countries. More than six million people were receiving ART in these countries at the end of 2010 [1,2]. As ART continues to expand in resource-limited settings, the emergence of HIV drug resistance mutations (DRMs) is challenging. Due to the absence of virological monitoring in routine clinical care together with the use of antiretroviral (ARV) drugs with low genetic barriers (such as 3TC and non-nucleoside reverse transcriptase inhibitors [NNRTIs]), concerns remain regarding the emergence of high-level resistance during first-line therapy. If ART regimens are not effectively delivered, DRMs could become widespread, leading to an increase in therapeutic failures, transmission of drug-resistant viruses, and a decrease in therapeutic options, treatment program effectiveness, and survival. Thus, as recommended by the World Health Organization (WHO), besides determining rates of transmitted DRMs, monitoring acquired DRMs emerging during treatment is crucial in resource-limited countries [3,4].

In Gabon (a country of 1.4 million inhabitants located in central Africa) where HIV-1 seroprevalence is high (approximately 6%) [5], ART became available in 2001 through the gradual implementation of ARV centres. To date, there is a network of 10 centres located throughout the nine provinces of the country [6]. Of the approximately 53,000 Gabonese subjects living with HIV-1, 10,000 were receiving ART by the end of 2009. Currently, ART is initiated when CD4 counts are ≤350 cells/µl, according to the WHO 2010 guidelines [7]. Due to financial and technical constraints, biological monitoring is restricted to CD4 counts, and remains associated with limited, if any, plasma HIV-1 RNA viral load (VL) measurements [8]. In Gabon, no study has yet reported the effectiveness of initial ART given through routine HIV care. Here we assessed virological failure (VF) rates and HIV DRMs among approximately 400 patients receiving ART for at least 12 months in two (semirural or rural) ARV centres in Gabon.

## Materials and Methods

### Study design and settings

According to the WHO recommendations [4], we conducted a cross-sectional study by performing a one-time HIV-1 RNA VL measurement free of cost to patients meeting the following criteria: (i) being confirmed HIV-1-positive; (ii) ≥18 years old; (iii) being on first-line ART for at least 12 months; and (iv) and consenting to participate in the study. The study was approved by the ethical biomedical research committee of Franceville (No. 023/2010/MESRS/CERB). After information about the study, an oral informed consent was obtained from all patients. All data were anonymized for further analysis.

Patients were consecutively enrolled from two HIV care centres. One ARV centre, located at Franceville (a city of 70,000 inhabitants), was a semirural site managing a total of approximately 1000 patients on ART. The second centre, located at Koulamoutou (a small town of 15,000 inhabitants) was a rural setting with approximately 400 treated subjects. A standardized form was filled out by medical doctors to record the following variables: sex, age, ARV use (date, name, switch), and previous CD4 counts obtained at ART initiation. Previous history of unstructured treatment interruptions (TIs) was also extracted from clinical records. Treatment interrupters were defined as subjects who defaulted and returned to care during their follow-up. When available, the duration of unplanned TIs was recorded. Patients who were transferred to other care centres were not considered as interrupters. If patients had a treatment interruption at the time of blood sampling, they were excluded from this survey.

### Laboratory testing

Whole blood specimens were collected in EDTA tubes at each centre. For Franceville, they were processed within six hours at the Centre International de Recherches Médicales de Franceville (CIRMF) Retrovirology Laboratory. After centrifugation, plasma was frozen at −80°C. For Koulamoutou, plasma specimens were processed similarly in the centre, stored at −20°C, and transported by road in a cool box once per week to the CIRMF Retrovirology Laboratory.

HIV seropositivity was confirmed using an in-house serotyping V3 immunoassay [9]. CD4 counts at enrolment were determined with flow cytometry (Fascount, Becton Dickinson, San Jose, CA). Plasma HIV-1 RNA levels were determined using the HIV Generic Viral Load assay (Biocentric, Bandol, France) [10]. This HIV-1 RNA test has a sensitivity threshold of 300 copies/ml by using 200 µl of plasma. Genotypic DR tests were carried out among viremic patients with VF (defined as a HIV-1 RNA viral load result ≥1000 copies/ml, according to the WHO 2010 recommendations) [11], using a previously described in-house assay [12]. ARV drug resistance mutations (DRMs) were identified and interpreted using the Agence Nationale de Recherches sur le SIDA (ANRS) algorithm (May 2011) (http://www.hivfrenchresistance.org). Mutations were also studied using the Stanford HIVdb genotypic resistance algorithm and then coded as major resistant mutations using the International AIDs Society (IAS) list from January 2012 (http://hivdb.stanford.edu). HIV-1 subtyping was performed by phylogenetic analysis of *pol* region sequences. Our laboratory participated in the 2011 quality control assessment of HIV-1 drug resistance sequencing implemented by the ANRS [13].

### Statistical analysis

All statistical analyses were performed using Stata 10™ software (Houston, TX). First, a comparison of patients’ characteristics between the two care centres was made using the χ^2^ for categorical variables and the Mann-Whitney-U test for continuous variables. Second, VF (≥1000 copies/mL) and DRM prevalence rates with 95% confidence intervals (95% CIs) were determined. Third, logistic regression models were used to examine the following explanatory variables as potential factors associated with VF: gender, age, centre, time on ART, unstructured TIs, and CD4 count at ART initiation (month 0) and at the time of VL assessment. The same seven variables plus HIV-1 RNA levels were further examined as predictors of at least one significant DRM among patients with VF. Finally, all variables with *P*-values <0.2 in univariate analyses were entered in multivariate analyses.

## Results

From March 2010 to March 2011, a total of 375 adult patients on first-line ART were consecutively included (Table 1). In both sites, more women were enrolled and most patients were thus women (74%). The median age of women was slightly lower than for men (40.8 years versus 47.7 years). Except one woman who tested positive to subtype O strain, all subjects harboured HIV-1 variants belonging to group M. A large majority (357/375, 95.2%) of patients were receiving two nucleoside reverse transcriptase inhibitors (NRTIs) plus one NNRTI (combination of D4T/AZT/ABC+3TC+NVP/EFV) whereas approximately 4% (n=14, including 11 women) started a protease inhibitor (PI)-based regimen. Four (approximately 1%) additional patients (including three women) were receiving TDF+3TC+EFV. The switch mainly concerned replacement of D4T by AZT or NVP by EFV. At ART initiation (M0), the overall median CD4^+^ count was 159 cells/µl (IQR, 70–248), with no significant difference between women (158 cells/µl) and men (164 cells/µl). At the time of enrolment, the median time on treatment was 33.6 months (range, 12–107) and was slightly higher for males (35.4 months) compared with females (32.3 months). The median CD4^+^ count reached 337 cells/µl (IQR, 215–492), with very similar values for women (334 cells/µl) and men (343 cells/µl). Previous unstructured TIs were recorded in more than 40% of patients (in women and men), with an overall median duration of seven months. There was no significant difference in the patients’ characteristics between the two care centres except the median time on ARV therapy, which was slightly longer in the semirural site than in the rural centre (*P=*0.03). Also unstructured TIs were more frequently recorded in patients from the semirural centre (approximately 46%) versus the rural site (approximately 33%) (*P=*0.05).

**Table 1:** Baseline characteristics of 375 HIV-1-positive patients treated with first-line ARV regimens in Gabon

Variables	Women (n=277)	Men (n=98)	Total (n=375^a^)
Age			
Median, years (IQR)	40.8 (34.5–46.9)	47.7 (41.5–53.2)	42.2 (35.8–48.9)
HIV V3 serotyping, n (%)			
HIV-1 M	266 (96.0)	93 (94.9)	359 (95.7)
HIV-1 O	1 (0.4)	0	1 (0.3)
HIV-1 N	0	0	0
HIV-1 P	0	0	0
HIV-2	0	0	0
Negative^b^	10 (3.6)	5 (5.1)	15 (4.0)
First-line ARV regimen, n (%)			
D4T/AZT/ABC+3TC+NVP/EFV^c^	263 (94.9)	94 (95.9)	357 (95.2)
2 NRTIs (D4T/AZT/DDI/3TC/ABC)+1 PI^d^	11 (4.0)	3 (3.1)	14 (3.7)
TDF+3TC+EFV	3 (1.1)	1 (1.0)	4 (1.1)
Time on ART (months)			
12–23	84 (30.3)	27 (27.6)	111 (29.6)
24–35	71 (25.6)	20 (20.4)	91 (24.3)
≥36	122 (44.1)	51 (52.0)	173 (46.1)
Median (IQR)	32.3 (20.6–49.7)	35.4 (21.3–50.9)	33.6 (21.2–50.4)
Unstructured treatment interruptions			
n (%)	122 (44.0)	40 (40.8)	162 (43.2)
Duration^e^, median (IQR) (months)	6 (2–13)	10 (6–21)	7 (3–15)
Recurrent events (n)	43/119 (36.1)	13/43 (30.2)	56/162 (34.6)
CD4^+^ count at ART initiation			
Median, cells/µl (IQR)	158 (76–249)	164 (65–247)	159 (70–248)
CD4^+^ count at enrolment			
Median, cells/µl (IQR)	334 (217–501)	343 (202–484)	337 (215–492)

a Including 300 patients from the semirural centre (Franceville) and 75 from the rural centre (Koulamoutou).

b The 15 samples found negative with the V3 serotyping HIV ELISA were found positive by HIV-1 western blot (New Lav Blot I, Bio-Rad, Marnes-La-Coquette, France).

c D4T(n=121/)/AZT(n=215)/ABC(n=21)+3TC+NVP(n=78)/EFV(n=279).

d IDV, n=6; NFV, n=4; LPV/r, n=4.

e The duration of unstructured TIs was recorded for 101 patients.

Abbreviations: ART, antiretroviral therapy; D4T, stavudine; ZDV, zidovudine; 3TC, lamivudine; NVP, nevirapine; EFV, efavirenz; IDV, indinavir; ABC, abacavir; DDI, didanosine; LPV/r, ritonavir-boosted lopinavir.

Overall, 171 (45.6%; 95% CI, 40.6–50.7) had a VL ≥300 copies/ml and were therefore defined as viremic on ART, with a median of 49,900 copies/ml (IQR, 5805–388,000). When using a threshold value of 400 copies/ml, 165 (44.0%; 95% CI, 39.0–49.1) subjects were viremic. For drug resistance assessment, genotypic tests were only done among the 155 (41.3%) viremic patients with a VL result ≥1000 copies/ml. Overall, 141/155 (91.0%) virus were successfully sequenced (ten could not be amplified and four had insufficient volume) and showed a very high HIV-1 genetic diversity. Although the predominant HIV-1 variants were CRF02_AG (n=46/141, 32.6%) and subtype A (n=23, 16.3%), 15 additional subtypes/CRFs (including subtypes G [n=11], D [n=7], C [n=3], H [n=3], F2 [n=1], J [n=1] and CRF37_cpx [n=9], CRF01_AE [n=5], CRF25_cpx [n=5], CRF11_cpx [n=3], CRF06_cpx [n=1], CRF09_cpx [n=1], CRF36_cpx [n=1], CRF43_02G [n=1], and CRF45_cpx [n=1]) and 19 mosaic unique recombinant forms (URFs) were also identified.

**Figure 1:**
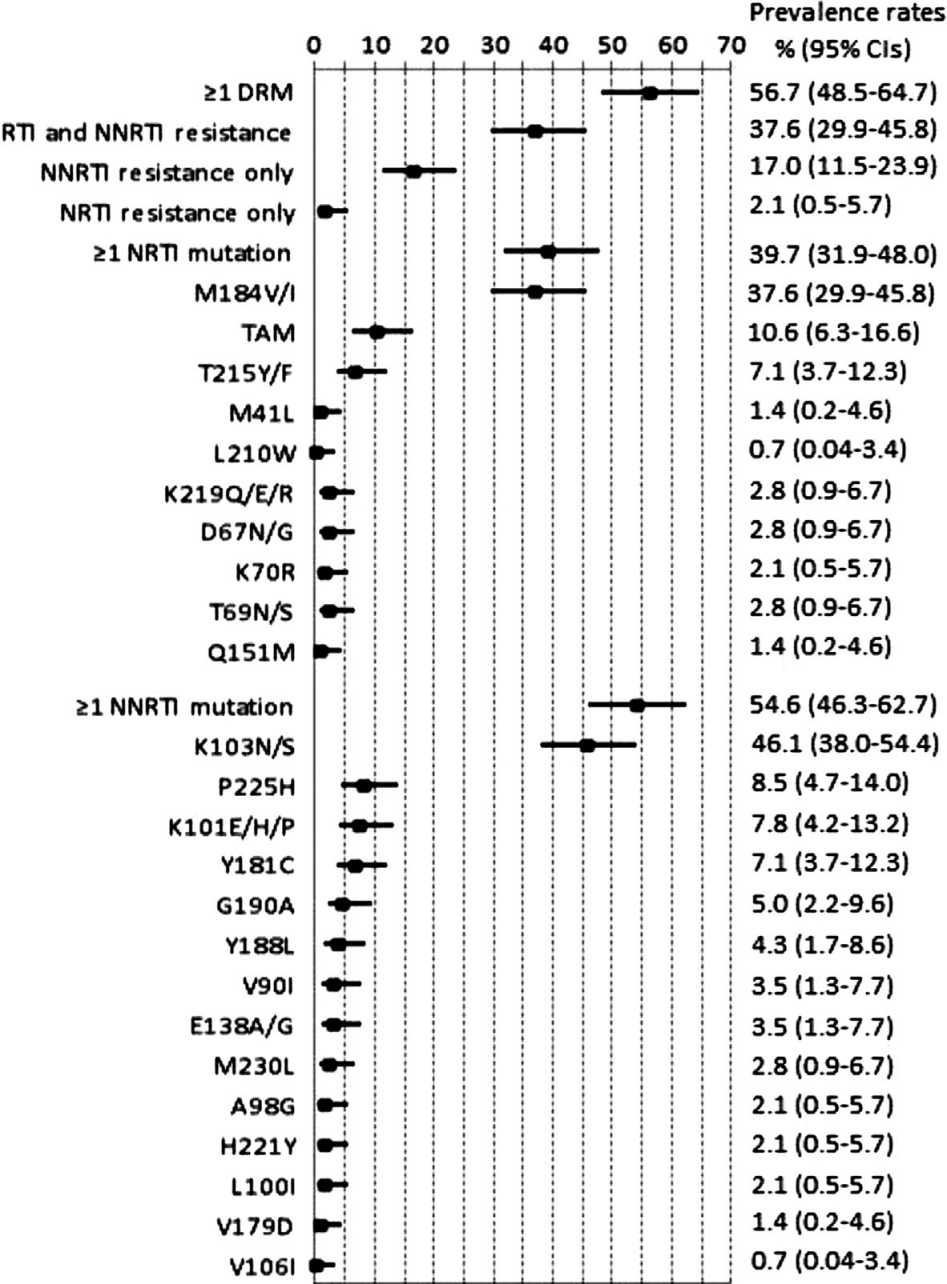
Frequency (forest plots) of drug resistance mutations in 141 viremic HIV-1-positive patients from Gabon and treated with first-line NNRTI-based antiretroviral therapy. Squares represent estimates and bars 95% confidence intervals (CIs). Abbreviations: NRTIs, nucleoside reverse transcriptase inhibitors; NNRTIs, nonnucleoside reverse-transcriptase inhibitors. M41L, L210W, and T215Y mutations are indicative of the TAM-1 pathway; D67N/G, K70R, T215F, and K219Q/E/R mutations are indicative of the TAM-2 pathway.

By using the ANRS algorithm, among the 141 sequenced specimens, 80 (56.7%) harboured ≥1 DRM (Figure 1). Overall, 53 (37.6%) showed dual-class resistance to NRTIs and NNRTIs, 24 (17.0%) harboured resistance to NNRTIs only, and three (2.1%) were only resistant to NRTIs. Around two-thirds of viral strains from patients with DRMs were resistant to at least two or three of the drugs constituting their ARV regimens. No clinically relevant protease resistance mutation was found amongst the four viremic patients with first-line PI-based regimens. Identical results were obtained using the Stanford HIVdb algorithm.

Of the 56 NRTI-associated DRMs, M184V/I selected by 3TC/FTC was the most frequent (n=53, 37.6%), followed by any thymidine analogue resistance TAM mutation (n=15, 10.6%). Ten patients had viruses with the TAM-1 mutation T215Y/F associated with AZT/D4T resistance. Other TAM-1 were M41L (n=2) and L210W (n=1). TAM-2 mutations included K219Q/E/R (n=4), D67N/G (n=4), and K70R (n=3). Three (3.7%) patients had viruses with at least three of these TAMs associated with AZT/D4T resistance. No patient harboured viruses having accumulated TAMs that confer also cross-resistance to ddI or ABC or TDF. No patient displayed viruses harbouring the K65R or K70E mutation selected by TDF. Two patients had HIV-1 strains harbouring the multi-NRTI resistance Q151M mutation associated with M184V/I and Y188L.

Among the 77 (54.6%) patients harbouring viruses resistant to at least one molecule of the NNRTIs class, the most commonly detected DRM was the K103N/S (n=65, 46.1%). Major NNRTIs DRMs were also obtained at positions P225H (n=12), K101E (n=11), Y181C (n=10), G190A (n=7), Y188L (n=6), V90I (n=5), E138A/G (n=5), M230L (n=4), A98G (n=3), H221Y (n=3), L100I (n=3), V179D (n=2), and V106I (n=1). In two patients treated with NVP, viruses were found resistant to etravirine (ETV), the second generation NNRTI drug, due to the simultaneous presence of the Y181C and H221Y mutations. Five additional viruses were predicted to be possibly resistant to ETV, including three viruses harbouring the E138A/G/Q/R DRM, one with the three DRMs K101E/H/I/P/R, Y181C, and G190A/S, and one with the three DRMs V90I, K101E/H/I/P/R, and G190A/S.

Independent factors associated with VF were being followed up at the semirural site (*P=*0.033), having experienced unstructured treatment interruptions (*P=*0.0044), and having low CD4^+^ counts at enrolment (*P*<0.0001) (Table 2). We found no significant association between VF and sex (*P=*0.24), age, or CD4 at M0. A longer time on ART (*P=*0.0008) and being followed up at the rural centre (*P=*0.021) were risk factors for DRMs. We found no significant association between the presence of DRMs and sex (*P=*0.95), age, or CD4 at M0. VL levels were not associated with DRM acquisition (Table 2).

**Table 2:** Risk factors associated with VF and DRMs among HIV-1-positive patients treated with first-line ARV regimens in Gabon

Variables	VF		P^a^	P^b^	DRM≥1		P^a^	P^b^
	
	VL<1000 cp/ml (N=220)	VL≥1000 cp/ml (N=155)			No (N=61)	Yes (N=80)		
Sex, n (%)			0.24	–			0.95	–
Male	63 (63.6)	36 (36.4)			14 (43.8)	18 (56.2)		
Female	157 (56.9)	119 (43.1)			47 (43.1)	62 (56.9)		
Age (years), median (IQR)	42.1 (36.8–50.2)	42.3 (35.3–47.9)	0.17	0.3	42.4 (34.4–47.0)	41.6 (35.8–48.0)	0.22	–
Time on ART (years), median (IQR)	32.9 (21.3–48.8)	33.1 (20.4–52.9)	0.4		24.1 (17.5–40.0)	42.4 (25.2–57.9)	0.012	0.00008
HIV care centre, n (%)			0.01	0.0033			0.083	0.021
Rural	54 (72.0)	21 (28.0)			5 (25.0)	15 (75.0)		
Semirural	166 (55.3)	134 (44.7)			56 (46.3)	65 (53.7)		
Unstructured treatment interruptions, n (%)			<0.0001	0.044			0.14	0.34
No	149 (70.0)	64 (30.0)			28 (50.9)	27 (49.1)		
Yes	71 (43.8)	91 (56.2)			33 (38.4)	53 (61.6)		
CD4^+^ count at ART initiation, median, cells/µl								
(IQR)	161 (69–250)	157 (80–240)	0.9	–	156 (94–245)	122 (54–237)	0.33	–
CD4^+^ count at enrolment, median, cells/µl								
(IQR)	412 (295–566)	229 (120–350)	<0.0001	<0.0001	232 (139–349)	213 (110–309)	0.42	–

HIV-1 RNA VL (log_10_ cp/ml), median (IQR)	–	–	–	–	5.14 (3.98–5.83)	4.98 (4.19–5.66)	0.74	–

a Univariate analysis

b Multivariate analysis.

Abbreviations: VL, viral load; VF, virological failure; DRM, drug resistance mutation; ART, antiretroviral treatment; IQR, interquartile range.

## Discussion

Here, for the first time in Gabon, we provided VF rates and DRM patterns in adult patients living with non-B HIV-1 strains and receiving mostly (>95%) first-line thymidine analogue, 3TC, and NNRTI-based ART for a median period of 34 months. Our survey, conducted in semirural and rural settings, first revealed a high rate of VF, with 41% of patients showing VL results ≥1000 copies/ml. In our survey, a history of unplanned treatment interruptions and CD4^+^ counts at inclusion were strong predictors of VF. Also, a higher VF rate was found for the semirural care centre. Second, among participants who virologically failed ART, 56.7% carried ≥1 DRM and almost 40% had dual-class resistance to NRTIs and NNRTIs. DRMs were consistent with expected patterns given first-line NNRTI-based therapy predominantly used in Gabon. The proportion of patients with DRMs significantly increased with time on ART and was higher for the rural site.

Comparison of VF (or success) and DRM profiles rates obtained in different African populations must be made cautiously because study design, VL assays and threshold used, and duration of ART at time of failure may vary. In a review, Barth and colleagues reported virological success (defined by a VL <400 cp/ml) rates in sub-Saharan Africa of 78%, 76%, and 67% after 6, 12, and 24 months of ART, respectively [8]. Compared with those pooled data, the success rates found herein by using an identical sensitivity threshold (<400 cp/ml) were consistent for the rural centre (68% for a median of 28 months of treatment) but potentially lower for the semirural centre (53.0% for a median of 35 months of treatment).

In addition, the burden of DRMs found in our survey was consistent with previous studies in which prevalence of DRMs showed highly contrasted results, with NNRTI mutations ranging from 47 to 100%, the M184V/I mutation from 24 to 81%, and any TAM from 0 to 63% [14–22]. However, in our study, it must be noted that patients from the semirural site had infrequent DR mutations, albeit with a higher rate of VF as mentioned here. One reason for explaining varying patterns of VF and drug resistance may be related to transient episodes of viremia due to patient-initiated unstructured treatment interruptions that are a reality of routine clinical care in African ART programs [23]. In our survey, they were more frequent at the semirural site in comparison with the rural centre. They implied patient-related factors (such as incomplete adherence, difficulties with payment for travelling to the HIV care centre and/or for routine biological tests, and toxicity and side effects of ART regimens), as well as program-related factors (such as pharmacy stock-outs). The impact of structured treatment interruptions (STIs) on virological outcomes has been previously reported through randomized STI trials [24–26]. For instance, in the Trivacan trial conducted in Ivory Coast, Danel *et al*. obtained 24% of drug-resistant viruses in a two-months-off, four-months-on ART arm, compared with 9% in a continuous strategy arm, with a highly significant difference for resistance to mostly NNRTIs. By contrast, very few studies “in real world settings” investigated the impact of unplanned treatment interruptions on the development of DRMs [23,27]. In our setting, we think that early transient viremia may occur prior to the accumulation of DRMs or may be associated with a reversion to wild type viruses. As a consequence, among patients having experienced unstructured treatment interruptions, resistance would not be identified as frequently.

Our study is limited by its cohort cross-sectional design and does not provide any information on the overall efficiency of the national ART program. Even if it is known that rates of lost to follow-up are higher in the first year and tend to decrease thereafter, no information was available on the dropout/death rates of the clinics and on the presence (or not) of DRM before ART initiation. In addition, subjects with a VL result ranged between 300 and 1000 copies/ml may harbour mutant HIV strains with DRMs. Therefore, the drug resistance rate that we obtained in our study is most likely a minimal estimate. Another limitation of our study is represented by the fact that no data on baseline prevalence of HIV drug resistance was available for our studied population. However, all studies recently conducted among untreated Gabonese subjects revealed low levels (≤1%) of transmitted HIV DR [5,28,29]. Finally, we were not able to fully investigate gender-related differences in virological outcomes given the relatively small sample size for men and the lack of information for a history of ARV prophylaxis to prevent mother-to-child transmission (MTCT) among women enrolled in our study.

## Conclusions

In summary, this is the first study conducted in Gabon providing VF rates and DRM patterns in adult patients receiving first-line ART. In sub-Saharan Africa, where NNRTI-based regimens are recommended as the standard of care for first-line ART, strengthening virological monitoring aimed at early failure detection, together with preventing unintended treatment interruptions, are a global public health priority.

Access to virological monitoring should be also increased to limit the level and complexity of drug resistance observed in non-B HIV-1 strains circulating in resource-limited settings. Increase in VL monitoring will prevent inappropriate switches and preserve susceptibility to second-line regimens in Africa [30–32]. As reported recently by Wallis and colleagues in South Africa [33], it is feasible to obtain low (≤10%) rates of VF and less complex drug resistance profiles using the D4T+3TC+EFV/NVP regimens.

Finally, the WHO/HIVResNet Global HIV drug resistance (HIVDR) prevention, surveillance, and monitoring strategy must be strengthened worldwide at sentinel sites to inform national health authorities on the efficiency of first-line ART and to allow recommendations on future ART strategies. Our study showed that, in the vast majority of our patients, the second-line regimen recommended by WHO would be active in our settings. Nevertheless, a minority showed complex drug resistance profiles predictive of resistance to the usual second-line regimens. Emerging multi-NRTI resistance in sub-Saharan Africa would not only compromise second-line treatment options and the success of antiretroviral roll-out but could also contribute to the spread of drug-resistant variants worldwide [34]. Thus frequent VL monitoring is definitively required to limit the threat of increased transmitted resistance in the face of continuing incident infection as observed in Gabon.

## References

[1] Lawn SD, Harries AD, Wood R (2010). Strategies to reduce early morbidity and mortality in adults receiving antiretroviral therapy in resource-limited settings. Curr Opin HIV AIDS.

[2] Barth RE, Tempelman HA, Moraba R, Hoepelman AI (2011). Long-Term Outcome of an HIV-Treatment Programme in Rural Africa: Viral Suppression despite Early Mortality. AIDS Res Treat.

[3] Bennett DE, Bertagnolio S, Sutherland D, Gilks CF (2008). The World Health Organization's global strategy for prevention and assessment of HIV drug resistance. Antivir Ther.

[4] Jordan MR, Bennett DE, Wainberg MA, Havlir D, Hammer S, Yang C (2012). Update on World Health Organization HIV drug resistance prevention and assessment strategy: 2004–2011. Clin Infect Dis.

[5] Caron M, Makuwa M, Souquiere S, Descamps D, Brun Vezinet F, Kazanji M (2008). Human immunodeficiency virus type 1 seroprevalence and antiretroviral drug resistance-associated mutations in miners in Gabon, central Africa. AIDS Res Hum Retroviruses.

[6] Ndong GP, Adam G, Mouala C, Faucherre V, Kouely PN, Sibeoni J, Courpotin C (2008). National coordination of the ambulatory treatment centers (ATC) in Gabon: a new process to conduct the scaling up of care for people living with HIV-AIDS. Sante.

[7] Walensky RP, Wood R, Ciaranello AL, Paltiel AD, Lorenzana SB, Anglaret X (2010). Scaling up the 2010 World Health Organization HIV treatment guidelines in resource-limited settings: a model-based analysis. PLoS Med.

[8] Barth RE, van der Loeff MFS, Schuurman R, Hoepelmon AIM, Wensing AMJ (2010). Virological follow-up of adult patients in antiretroviral treatment programmes in sub-Saharan Africa: a systematic review. Lancet Infect Dis.

[9] Vallari A, Holzmayer V, Harris B, Yamaguchi J, Ngansop C, Makamche F (2011). Confirmation of putative HIV-1 group P in Cameroon. J Virol.

[10] Liégeois F, Boué V, Mouinga-Ondémé A, Kenfack D, Sica J, Rouet F (2012). Suitability of an open automated nucleic acid extractor for high-throughput plasma HIV-1 RNA quantitation in Gabon (central Africa). J Virol Methods.

[11] World Health Organization (2010). Towards universal access: scaling up the priority HIV/AIDS interventions in the health sector. http://www.who.int/hiv/pub/2010progressreport/summary_en.pdf.

[12] Vergne L, Diagbouga S, Kouanfack C, Aghokeng A, Butel C, Laurent C (2006). HIV-1 drug-resistance mutations among newly diagnosed patients before scaling-up programmes in Burkina Faso and Cameroon. Antivir Ther.

[13] Descamps D, Delaugerre C, Masquelier B, Ruffault A, Marcellin AG, Izopet J (2006). Repeated HIV-1 resistance genotyping external quality assessments improve virology laboratory performance. J Med Virol.

[14] Ramadhani HO, Thielman NM, Landman KZ, Ndosi EM, Gao F, Kirchherr JL (2007). Predictors of incomplete adherence, virologic failure, and antiviral drug resistance among HIV-infected adults receiving antiretroviral therapy in Tanzania. Clin Infect Dis.

[15] Marconi VC, Sunpath H, Lu Z, Gordon M, Koranteng Apeagyei K (2008). Prevalence of HIV-1 drug resistance after failure of a first highly active antiretroviral therapy regimen in KwaZulu Natal, South Africa. Clin Infect Dis.

[16] Barth RE, Wensing AM, Tempelman HA, Moraba R, Schuurman R, Hoepelman AI (2008). Rapid accumulation of nonnucleoside reverse transcriptase inhibitor-associated resistance: evidence of transmitted resistance in rural South Africa. AIDS.

[17] Hosseinipour MC, van Oosterhout JJ, Weigel R, Phiri S, Kamwendo D, Parkin N (2009). The public health approach to identify antiretroviral therapy failure: high-level nucleoside reverse transcriptase inhibitor resistance among Malawians failing first-line antiretroviral therapy. AIDS.

[18] Dlamini JN, Hu Z, Ledwaba J, Morris L, Maldarelli FM, Dewar RL (2011). Genotypic resistance at viral rebound among patients who received lopinavir/ritonavir- or efavirenz-based first antiretroviral therapy in South Africa. J Acquir Immune Defic Syndr.

[19] Charpentier C, Talla F, Nguepi E, Si-Mohamed A, Belec L (2011). Virological failure and HIV type 1 drug resistance profiles among patients followed-up in private sector, Douala, Cameroon. AIDS Res Hum Retroviruses.

[20] Dagnra AY, Vidal N, Mensah A, Patassi A, Aho K, Salou M (2011). High prevalence of HIV-1 drug resistance among patients on first-line antiretroviral treatment in Lome, Togo. J Int AIDS Soc.

[21] Muwonga J, Edidi S, Butel C, Vidal N, Monleau M, Okenge A (2011). Resistance to antiretroviral drugs in treated and drug-naive patients in the Democratic Republic of Congo. J Acquir Immune Defic Syndr.

[22] Hamers RL, Sigaloff KC, Wensing AM, Wallis CL, Kityo C, Siwale M (2012). Patterns of HIV-1 drug resistance after first-line antiretroviral therapy (ART) failure in 6 sub-Saharan African countries: implications for second-line ART strategies. Clin Infect Dis.

[23] Kranzer K, Lewis JJ, Ford N, Zeinecker J, Orrell C, Lawn SD (2010). Treatment interruption in a primary care antiretroviral therapy program in South Africa: cohort analysis of trends and risk factors. J Acquir Immune Defic Syndr.

[24] Yerly S, Fagard C, Gunthard HF, Hirschel B, Perrin L (2003). Drug resistance mutations during structured treatment interruptions. Antivir Ther.

[25] Ruiz L, Paredes R, Gomez G, Romeu J, Domingo P, Perez Alvarez N (2007). Antiretroviral therapy interruption guided by CD4 cell counts and plasma HIV-1 RNA levels in chronically HIV-1-infected patients. AIDS.

[26] Danel C, Moh R, Chaix ML, Gabillard D, Gnokoro J, Diby CJ (2009). Two-months-off, four-months-on antiretroviral regimen increases the risk of resistance, compared with continuous therapy: a randomized trial involving West African adults. J Infect Dis.

[27] Luebbert J, Tweya H, Phiri S, Chaweza T, Mwafilaso J, Hosseinipour MC (2012). Virological failure and drug resistance in patients on antiretroviral therapy after treatment interruption in Lilongwe, Malawi. Clin Infect Dis.

[28] Mintsa Ndong A, Caron M, Plantier JC, Makuwa M, Le Hello S, Courgnaud V (2009). High HIV type 1 prevalence and wide genetic diversity with dominance of recombinant strains but low level of antiretroviral drug-resistance mutations in untreated patients in northeast Gabon, central Africa. AIDS Res Hum Retroviruses.

[29] Caron M, Etenna Lekana-Douki S, Makuwa M, Obiang-Ndong GP, Biba O, Nkoghe D (2012). Prevalence, genetic diversity and antiretroviral drugs resistance-associated mutations among untreated HIV-1-infected pregnant women in Gabon, central Africa. BMC Infect Dis.

[30] Gupta RK, Hill A, Sawyer AW, Cozzi-Lepri A, von Wyl V, Yerly S (2009). Virological monitoring and resistance to first-line highly active antiretroviral therapy in adults infected with HIV-1 treated under WHO guidelines: a systematic review and meta-analysis. Lancet Infect Dis.

[31] Sigaloff KC, Hamers RL, Wallis CL, Kityo C, Siwale M, Ive P (2011). Unnecessary antiretroviral treatment switches and accumulation of HIV resistance mutations; two arguments for viral load monitoring in Africa. J Acquir Immune Defic Syndr.

[32] Sigaloff KC, Ramatsebe T, Viana R, de Wit TF, Wallis CL, Stevens WS (2012). Accumulation of HIV drug resistance mutations in patients failing first-line antiretroviral treatment in South Africa. AIDS Res Hum Retroviruses.

[33] Wallis CL, Papathanasopolous MA, Fox M, Conradie F, Ive P, Orrell C (2012). Low rates of nucleoside reverse transcriptase inhibitor resistance in a well-monitored cohort in South Africa on antiretroviral therapy. Antivir Ther.

[34] Hamers RL, Wensing AM, Back NK, Arcilla MS, Frissen JP (2011). Multi-nucleoside reverse transcriptase inhibitor resistant HIV type-1 in a patient from Sierra Leone failing stavudine, lamivudine and nevirapine. Antivir Ther.

